# Engineering spherical nucleic acids for precision cancer therapy: design strategies and medical applications

**DOI:** 10.7150/thno.132738

**Published:** 2026-06-17

**Authors:** Yilin Wang, Wenbing Wu, Fuli Yao

**Affiliations:** 1 School of Basic Medical Sciences, Southwest Medical University, Luzhou, Sichuan, China.; 2 Department of Biochemistry and Molecular Biology, School of Basic Medical Sciences, Southwest Medical University, Luzhou, Sichuan, China.

**Keywords:** spherical nucleic acid, cancer therapy, biomaterial, targeted delivery, immunotherapy

## Abstract

Malignant tumor treatment still faces issues like insufficient targeting, drug resistance, and immunosuppression. Spherical nucleic acids (SNAs), with their three-dimensional core-shell structure and densely packed, radially oriented oligonucleotide shell, enable transfection-free cellular uptake and provide nuclease resistance and stability. This review examines SNA engineering strategies and their impact on precision oncology. Functionalization with antibodies, aptamers, or antisense oligonucleotides enables SNAs to target key molecules such as human epidermal growth factor receptor 2 (HER2), programmed death-ligand 1 (PD-L1), and toll-like receptors (TLRs). Advances in stimuli-responsive, self-assembled, liposomal, and peptide-based carrier systems facilitate controlled drug release and modulation of the tumor microenvironment (TME). Diagnostic applications of SNAs include electrochemical, fluorescent, and colorimetric sensing systems for detecting biomarkers such as exosomes, miRNAs, alpha-methylacyl-CoA racemase (AMACR), and telomerase. Therapeutically, SNAs co-deliver chemotherapeutics and immunoadjuvants, support cancer vaccines, and exert efficacy in various tumors, including the central nervous, reproductive, digestive, hematological, barrier, and respiratory systems. Early clinical studies indicate a favorable biosafety profile, but issues remain regarding delivery efficiency, target selectivity, scalability, and long-term safety. Progress toward biodegradable, machine-learning-guided SNA platforms may soon make this nanotechnology a fundamental part of personalized, precision cancer medicine.

## Introduction

Malignant tumors pose a threat to human health, and there are problems such as poor targeting specificity, drug resistance, and immunosuppressive tumor microenvironment (TME) during treatment 1-8. Spherical nucleic acids (SNAs) are prepared by densely modifying the surface of nanoparticles with oligonucleotide shells 9-13, which have a three-dimensional core-shell structure, and their physicochemical properties and biological modes of action are significantly different from those of linear nucleic acids. This structure can enable cellular uptake via the endocytic pathway, mediated by the non-classical scavenger receptor class A (SR-A), without the need for transfection reagents; significantly improves nuclease resistance; and thus can serve as an ideal platform for safe and efficient nucleic acid drug delivery 14-18. Such technologies have the potential to overcome the limitations of traditional therapies; the systematic organization of the structural design, mechanistic analysis, and translational research has considerable research value.

The development of SNA nanostructures actually has a connected path, including conceptual updates, clarification of underlying mechanisms, and functional design, which complement each other. At present, groundbreaking research in academia on this type of structural configuration has confirmed that when oligonucleotides on the surface of nanoparticles are arranged radially and densely stacked, new properties will be derived. For example, it can significantly enhance the resistance to nucleases, and there will also be synergistic hybridization effects, while the efficiency of cellular uptake will also be significantly increased. This also makes SNAs a type of structurally unique therapeutic carrier, no longer just a passive delivery tool 19, 20. To unleash this potential, it is necessary to first understand how these structures regulate the process of cell entry. The density, orientation, and curvature of the shell all have an impact on the uptake of SNA. SNA is mainly internalized by cells through the endocytic pathway mediated by SR-A, which has been confirmed by relevant systematic studies 21, 22, providing a reasonable basis for engineering research on targeted functions. Now it is possible to directly incorporate molecular recognition into the SNA framework to complete related designs, such as binding antibodies to tumor receptors like human epidermal growth factor receptor 2 (HER2), using antisense oligonucleotides (ASOs) to suppress immune checkpoints like programmed death-ligand 1 (PD-L1), and integrating aptamers to achieve high-affinity recognition. The scope of related research is in 23-25, which also indicates that specific programming operations can be achieved without compromising the integrity of the structure itself. Based on this targeting direction, various modifications have been made to the nanocarrier core using enzyme-responsive release systems, metal coordination platforms, liposome structures, and peptide scaffolds 26-31. A modular design approach has also been developed, separating the outer shell responsible for targeting and immune regulation from the inner core responsible for drug loading and maintaining structural stability. This approach can also be applied in clinical settings 32-38. However, it is not yet fully understood whether this modular feature can stably produce reproducible drug metabolism effects in different tumor subtypes.

The functional scope of SNAs has long exceeded gene silencing and drug delivery and now covers cancer immunotherapy, vaccine development, and molecular detection 39. Multivalency and surface-oriented spatial arrangement are the common structural rules of these three application fields. In immunotherapy, the high-density, geometrically constrained presentation of immunostimulatory nucleic acids, rather than their inherent sequence activity, determines immunological efficacy and cell type selectivity. For example, hierarchical structures can activate both TLR3 and TLR9 40, while high-density RNA shells can selectively bind to plasmacytoid dendritic cells via TLR7/8 and remodel the suppressive TME 41. Vaccine design extends this logic to the coordinated co-display of antigens and adjuvants, where spatial location and surface density can regulate adaptive immune responses. The delivery of antigens and co-stimulatory molecules can induce potent antitumor memory 42, as well as the precisely tuned antigen-anchoring effect in dual-antigen structures 43, collectively demonstrating that surface arrangement can serve as a programmable means of regulating vaccine efficacy. In the field of diagnosis, the same high-density nucleic acid layers can amplify signal transduction through multivalent hybridization and maintain colloidal stability in complex matrices, supporting detection platforms such as electrochemical biosensing, fluorescence imaging, extracellular vesicle recognition, and miRNA quantification 44-47. These detections involve biomarkers including alpha-methylacyl-CoA racemase (AMACR) and telomerase. Clinical translation faces challenges in delivery, production, and biosafety, but human studies have laid a safety foundation 48, 49. Many immunostimulatory properties may also make nucleic acid nanostructures trigger off-target innate immune activation, requiring dose-response and biodistribution tests.

**Figure 1:**
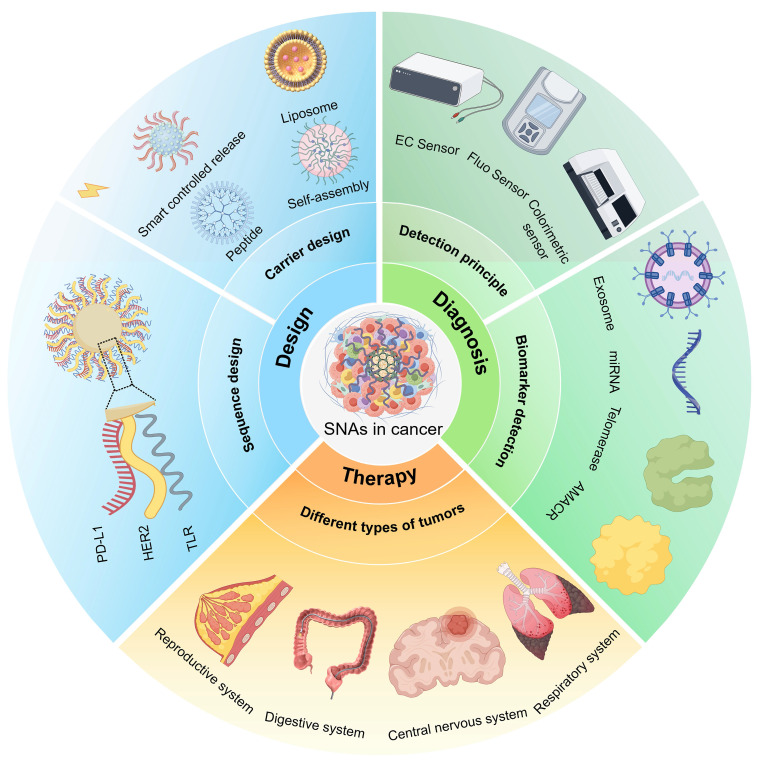
Schematic of SNA engineering for precision cancer therapy, illustrating sequence design, carrier construction, diagnostic biomarker detection, and therapeutic deployment across diverse cancers. Abbreviations: AMACR: alpha-methylacyl-CoA racemase; EC: electrochemical; Fluo: fluorescent; HER2: human epidermal growth factor receptor 2; PD-L1: programmed death-ligand 1; TLR: toll-like receptor.

This review systematically summarizes the core structural features and biological basis of SNAs as an innovative platform for cancer therapy, as shown in Figure 1. It also covers the design of targeting sequences, diverse nanocarrier construction methods, and related content on intelligent delivery systems. It analyzes the relevant design principles and the latest research progress. Subsequently, the specific applications of SNAs in drug delivery, vaccine development, disease diagnosis, and multi-system tumor therapy are discussed. At the same time, the current status of clinical safety evaluation and future research directions are summarized to provide references for subsequent related research and clinical translation.

## 1. Structural and functional characteristics of SNAs

### 1.1. Structure

The defined structural features of SNAs are a three-dimensional, radiating, and orderly nucleic acid shell, in which oligonucleotides are densely packed and project outward from the nanoparticle core in a unified direction 9, 10, 14, 50, 51. This geometric shape gives rise to an extraordinary molecular packing density and also reorganizes intermolecular electrostatic and hydrophobic interactions within the shell 15. The architectural features of SNA that are determined by constraints on three-dimensional space rather than by nucleic acid sequences are as described in 52, 53. In all SNA variants, the geometric foundation has generated a unified pattern of densely arranged radial strands 54.

The core components that act as geometric templates for oligonucleotide ligation and as chemical anchors are defined by gold nanoparticles functionalized via gold-thiol chemistry, forming the initial SNA platform 22, 55. Since then, the expanded platform has incorporated silver, silicon dioxide, iron oxide, quantum dots, polymers, liposomes, proteins, and other inorganic and organic materials 56-58, thereby creating a series of core-shell composite structures with adjustable size, curvature, and surface chemical properties.

A high-density layer consists of DNA, RNA, or chemically modified nucleic acid chains 59. The organization of this layer into single- or multi-layer structures is determined by the length, sequence, and spatial distribution of the nucleic acid chains 60. The interfacial chemistry that connects the core and the shell, silane coupling, amide bonding, and click reactions, determines the interchain spacing and three-dimensional crowding within the corona 61.

### 1.2. Function

The functional scope of SNAs stems directly from the high-density, radially arranged nucleic acid shells, which confer biological properties that are either significantly enhanced relative to linear oligonucleotides or absent in linear oligonucleotides 17, 18, 62-64. These properties, including the ability to enter cells without transfection, multivalent targeted binding, resistance to enzymatic digestion, and immunomodulatory activity, are relevant to tumor treatment and central nervous system diseases 21, 40, 48, 65, 66.

Different types of mammalian cells can take up nucleic acids through a transfection-free pathway without the need for cationic liposomes or polymer carriers 17. Specific recognition between the compact nucleic acid shell and scavenger receptors on the cell membrane drives clathrin-mediated endocytosis. SNA with a C_60_ core and 12 radially arranged DNA strands show 400-1000-fold higher cellular uptake efficiency by MCF-7 cells than free DNA after 6 h of treatment at a concentration of 0.5 μM, as measured by flow cytometry. The effective intracellular concentration of protein-based SNAs is approximately 4 orders of magnitude lower than that of free proteins, and they remain functional at 100 pM. In contrast, conventional protein transfection typically requires micromolar concentrations 67, 68.

In addition to absorption, the multivalent display of these chains can, to some extent, support the cooperative binding with surface receptors and biomolecular targets, which is something that a single linear oligonucleotide can scarcely achieve easily. Immune signaling provides a particularly clear demonstration of this principle, because many pattern-recognition receptors require ligand-induced clustering to initiate downstream cascades. Densely arrayed immune-activating sequences on SNAs promote TLR clustering on the cell surface and elicit responses far stronger than those triggered by free nucleic acids 66, whereas sequence micro-tuning of the identical platform switches the immune response from activation to suppression 69.

Enzymatic resistance and tunable circulation jointly define the pharmacokinetic profile of SNAs. Steric crowding and electrostatic repulsion within the densely packed corona impair nuclease binding and act as a dynamic protective layer 70, reducing nuclease-mediated clearance and prolonging systemic exposure. Liposomal SNAs bearing high-affinity diacylglycerol anchors extend the serum half-life of surface-immobilized DNA more than 20-fold relative to cholesterol-anchored constructs 58, and the circulation half-life of RNA-functionalized gold nanoparticle SNAs in mice ranges from approximately 1 min under minimal poly(ethylene glycol) (PEG) loading to approximately 10 min at high PEG surface density 10.

## 2. Design of engineered SNAs

The design concept of SNA is fundamentally different from that of other nucleic acid-based nanotechnologies. In lipid nanoparticles (LNPs), nucleic acids are passively encapsulated within the lipid core-shell structure; the lipid envelope determines the biological interface, and therapeutic activity depends on intracellular release following lipid-mediated uptake 71-73. DNA origami uses methods to create different structures; here, hundreds of staple strands fold a long single-stranded scaffold into geometrically precise nanoscale architectures 74-76, and therapeutic agents, targeting ligands, and imaging probes are site-specifically attached as exogenous cargo to these pre-assembled scaffolds 77. Although this strategy can attain nanoscale spatial addressability, the nucleic acid framework itself is structurally passive, neither driving receptor-mediated cell entry nor initiating endosomal immune signals.

SNAs work distinctly. The tightly packed, radially arranged oligonucleotide shell serves as the primary bioactive interface rather than a structural framework or delivery vehicle. This architecture enables transfection agent-free cellular uptake 21, 63, activates endosomal TLR9 with up to 80-fold greater potency (EC₅₀) than free phosphodiester oligonucleotides in RAW-Blue macrophages *in vitro*, an effect that is backbone- and incubation time-dependent 41, 66, and confers intrinsic nuclease resistance through steric shielding while mediating gene silencing 63, 78. Cellular recognition, immune activation, and gene regulation thus converge within a single nucleic acid corona, embodying a carrier-is-the-drug paradigm that has no analog in LNP or DNA origami systems.

This integrated bioactivity imposes a coupled design logic in which sequence composition, surface oligonucleotide density, and core-shell architecture must be co-optimized as a unified system rather than treated as independent parameters 43, 79. The design of SNAs is therefore pivotal to their diagnostic and therapeutic performance in oncology 80-83. Sequence design targets HER2, PD-L1, TLR9, and related molecules via antibody coupling, antisense nucleic acids, siRNA, and aptamers to achieve precise targeting and immune modulation. Carrier design encompasses intelligent controlled-release mechanisms, *in situ* self-assembly, liposomal core-shell bifunctionality, and structural–functional peptide integration.

### 2.1. Design of SNA sequences

#### 2.1.1. HER2

HER2 overexpression in breast cancer and related malignancies makes it an excellent molecular target for precise SNA design 84-86. It is not only a cell-surface antigen but also a signaling node, enabling the SNA platform to achieve selective cell uptake and targeted gene regulation.

**Figure 2:**
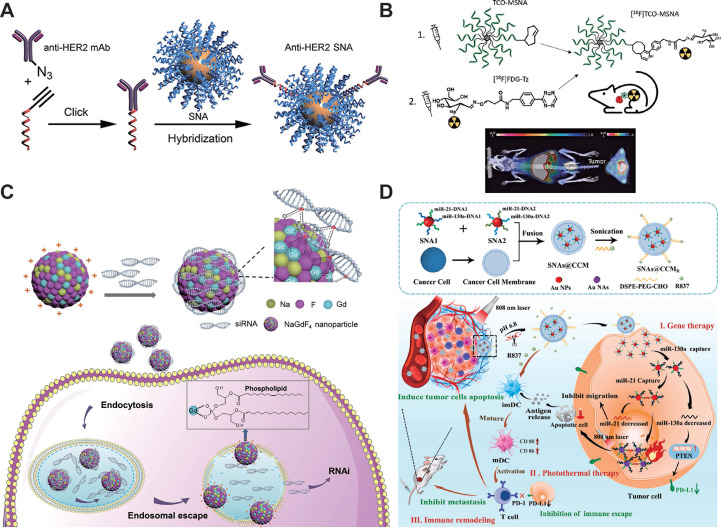
Design of SNA sequences. (A) Synthesis of anti-HER2 SNAs. Adapted with permission from 23, copyright 2012 American Chemical Society. (B) Schematic illustration of pretargeted PET imaging of TCO-functionalized SNAs via bioorthogonal tetrazine ligation. Adapted with permission from 25, copyright 2023 The Authors. (C) Schematic illustration of the interaction of NaGdF_4_ NPs with siRNA and the escape process of siRNA-functionalized NaGdF_4_ NPs from the endosome. Adapted with permission from 91, copyright 2022 Tsinghua University Press. (D) SNAs@CCM nanoparticles utilize overexpressed oncogenes in tumor cells to achieve on-demand photothermal therapy, reshape the immunosuppressive microenvironment, and efficiently eliminate primary and metastatic tumors. Adapted with permission from 97, copyright 2024 Elsevier Ltd.

Precision SNAs have been engineered as carriers that can act on HER2 at both the protein and transcription levels. Specific recognition of HER2-overexpressing cells can be achieved through non-covalently assembled antibody-DNA conjugates. Zhang *et al.* 23 illustrated this design concept in Figure 2A, which transforms the originally non-selective SNAs into precisely targeted delivery tools. Chen *et al.* 24 designed a β-cyclodextrin-modified supramolecular SNA carrying an HER2 antisense sequence at the mRNA level. This specific SNA can selectively inhibit HER2 protein expression and induce apoptosis in cells through the PI3K/Akt pathway.

The fidelity of real-time *in vivo* targeting has been verified using a quantitative imaging strategy, and relevant research has been conducted. A molecular SNA based on C_60_ and labeled with F-18 was designed by Äärelä *et al.* 87. It showed extended blood circulation and selectively accumulated in tumors of HER2-positive xenografts, achieving a tumor-to-muscle ratio of 2 on PET/CT imaging. Auchynnikava *et al.* 25 have developed SNAs functionalized with trans-cyclooctene (TCO) that target HER2 mRNA and have combined a pre-targeting PET strategy with [F-18]FDG-TZ to confirm precise enrichment in HER2-expressing tumors, as shown in Figure 2B.

Future efforts should prioritize optimizing covalent coupling methods to enhance the stability of antibody-nucleic acid complexes and systematically evaluate how specific chemical modifications govern nuclease resistance and biodistribution, both crucial steps towards the clinical translation of HER2-targeted, precise SNAs.

#### 2.1.2. PD-L1

PD-L1 enables tumors to evade immune surveillance and suppresses the antitumor response of T cells through its interaction with PD-1 88-90. SNAs possess considerable structural flexibility, allowing them to precisely match different nucleic acid forms to the various regulatory requirements of PD-L1 signaling.

In the context of gene silencing, the core design challenge lies in the efficient delivery of siRNA into cells. As shown in Figure 2C, by linking PD-L1-targeting siRNA to lanthanide-doped NaGdF₄ nanoparticles via a gadolinium phosphate backbone, the nanoparticles were endowed with membrane-binding capabilities, enabling them to disrupt endosomal structures and achieve sustained gene silencing, thereby inhibiting tumor growth in CT26 and 4T1 models 91. In another approach, Chou *et al.* 92 designed lipid-based small-molecule SNAs and loaded them with antisense DNA sequences that can directly block the PD-1/PD-L1 signaling pathway through gene regulation. This system can precisely reduce PD-L1 expression in various immune-related cell types within the TME, including tumor cells, dendritic cells, and myeloid suppressor cells. In the MC38 colorectal cancer model, this precisely coordinated downregulation enhances the recruitment and activity of CD8^+^ T cells, inhibits tumor progression, and prolongs survival.

There is a clear logical basis for PD-L1 serving as both a therapeutic target and a tumor-specific stimulator. Wei *et al.* 93 modified mesoporous hafnium oxide nanoparticles with PD-L1 aptamers and encapsulated methylene blue, thereby linking target recognition directly to imaging-driven activation. Selective drug delivery to PD-L1-high lesions achieved a tumor-to-background signal ratio of 7.97 ± 0.76, with drug retention exceeding 48 h. Combination with radiotherapy can further optimize the therapeutic effect, and no obvious systemic toxic reactions were observed.

siRNA, ASOs, and aptamers can interfere with PD-L1-mediated immunosuppression by targeting different mechanisms of action under a shared SNA adaptation framework. Integrating gene-silencing technology and real-time imaging detection into a single platform can facilitate the translation of precise tumor immunotherapy into systemic clinical applications.

#### 2.1.3. TLR

The core advantage of using SNAs for TLR agonist delivery lies in their ability to directly encode spatial and cell specificity into the nanostructure itself 94-96. As reported by Huang *et al.* 40, precisely designed compartmentalized structures physically separate different agonists between the shell and core, allowing the same antigen-presenting cell to activate both TLR3 and TLR9 simultaneously while maintaining the expression of co-stimulatory molecules and MHC-II molecules required for adaptive immune priming. Guan *et al.* 41 showed that precise regulation of shell components can independently determine cell tropism. RNA-coated SNAs are preferentially taken up by the TLR7/8-expressing cell population, dominated by plasmacytoid dendritic cells, and their pathway activation effect is much stronger than that of free RNA or lipid complex-coated formulations.

This structural design relies on responsive linking groups to chemically extend into the TME, achieving spatiotemporally controlled drug release. As shown in Figure 2D, Chen *et al.* 97 used acid-sensitive Schiff base bonds to conjugate the TLR7 agonist R837 to the surface of nanoparticles, enabling R837 release only in the acidic intratumoral environment. The miRNA-targeting core hybridizes to overexpressed oncogenic mRNA in tumor cells, thereby suppressing PD-L1 expression. Through this dual mechanism, innate immune activation and checkpoint blockade are synchronized. The SNAs@CCM system, therefore, achieves on-demand photothermal therapy, reshapes the immunosuppressive microenvironment, and efficiently eliminates both primary and metastatic tumors.

With these convergent design concepts, SNAs have emerged as carriers capable of integrating hierarchical TLR co-stimulation, shell-mediated cell selectivity, and TME-responsive agonist release within a single coherent structure. The molecular mechanisms by which dual TLR3/9 co-activation sustains co-stimulatory signaling remain unclear; however, quantitative models linking TLR activation kinetics to the intensity of downstream immune responses are essential for the rational optimization of SNA-based combination immunotherapies.

### 2.2. Design of SNAs' structural and functional architecture

#### 2.2.1. Intelligent controlled release carriers

Precise spatiotemporal control of drug release is a major advantage of SNAs as smart delivery vehicles. Unlike conventional nanoparticles, SNAs combine structural programmability with stimulus responsiveness, thereby tightly coupling payload release with pathological signals in the TME 33, 98-100.

**Figure 3:**
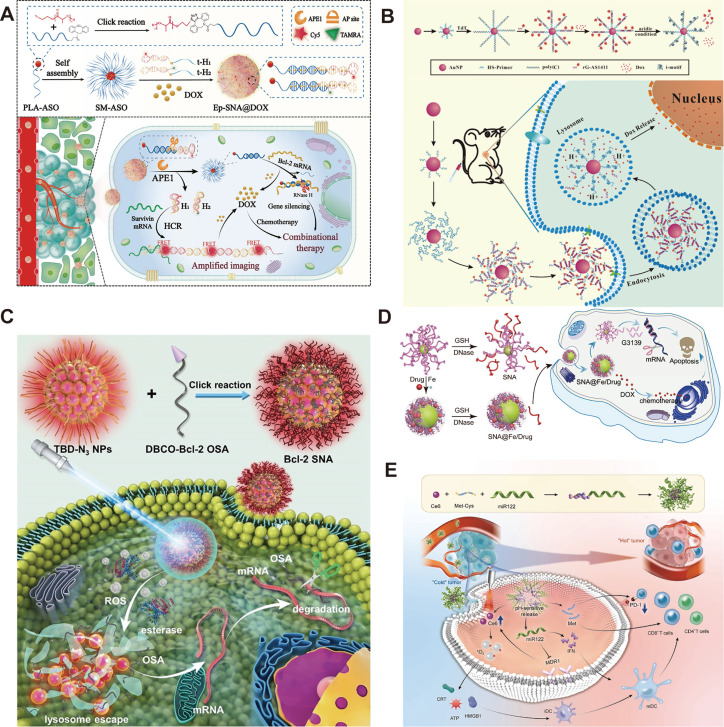
Structural and functional design of SNAs. (A) Construction and response mechanism of endogenous enzyme-activatable Ep-SNA@DOX for spatiotemporally controlled highly sensitive survivin mRNA imaging and combinational therapy in tumors. Adapted with permission from 26, copyright 2023 American Chemical Society. (B) Illustration of the preparation of DOX-loaded AuNP@Primers/(CG)n/AS1411s nanocarrier and its cancer cell-targeted delivery, internalization, and pH-responsive drug release. Adapted with permission from 101, copyright 2019 American Chemical Society. (C) Preparation of AIE PS-based Bcl-2 SNA, schematic representation of Bcl-2 SNA being taken up by tumor cells. Adapted with permission from 102, copyright 2020 Wiley. (D) The schematic illustration of the metal-coordinated SNA nanoplatform demonstrates its facile self-assembly and enhanced resistance to biothiols and nucleases, thereby enabling efficient drug delivery in tumor therapy. Adapted with permission from 28, copyright 2024 American Chemical Society. (E) Schematic illustration of a biodegradable peptide-core SNA nanoplatform co-delivering Ce6 and miR-122. Adapted with permission from 31, copyright 2024 Elsevier Inc.

Endogenous biochemical signals provide the most tumor-selective trigger mechanism for precise SNA-mediated release. As reported by Zeng *et al.* 26, the apurinic/apyrimidinic (AP) endonuclease 1, which is overexpressed in tumor cells, precisely cleaves the hairpin structure containing the AP site grafted onto the SNA, thereby simultaneously releasing the ASO and doxorubicin (DOX), as shown in Figure 3A. The same enzymatic specificity underpins SNA structures integrated with aptamers, which selectively shuttle the oncogenic RelA protein between cellular compartments in malignant cells while remaining structurally inert in normal tissues where enzyme levels are insufficient 27. Molecular recognition refines the mode of action of endogenous signaling. Li *et al.* 34 constructed ATP-responsive SNA structures. Upon binding to intracellular ATP, these structures undergo conformational changes according to predefined rules, and their on-demand release of substances aligns perfectly with the unique metabolic characteristics of tumor cells.

The physicochemical gradients within the TME provide an additional dimension of control for the precise release of payloads. The acidic environment of organelles drives the transition of double-stranded DNA to triple-stranded DNA via Hoogsteen base pairing, thereby combining precise lysosomal targeting with accurate pH-dependent DOX release 32. As described by Sun *et al.* 101, i-Motifs formed within densely packed DNA shells also trigger explosive payload release under acidic conditions, as shown in Figure 3B. Light exposure serves as the external trigger for precise release. As shown by Shi *et al.* 102, photosensitizer-modified SNAs generate singlet oxygen upon light exposure, disrupting the lysosomal membrane and facilitating the precise escape of nucleic acids into the cytoplasm, as shown in Figure 3C.

Despite its wide range of applications, there is still one key limitation. The mechanism underlying the coordination of the release kinetics of multiple therapeutic components on a single SNA carrier platform remains unclear, and the time lag between gene silencing and chemotherapy may weaken rather than enhance synergistic effects. Multi-responsive nanoparticles capable of sequential, programmed release represent a key research direction for advancing precise cancer therapy.

#### 2.2.2. Self-assembly

Self-assembly is not merely a convenient method for synthesis; it is a construction logic that enables the precise design of SNAs 35, 103, 104. Using sequence-specific DNA hybridization technology, the surface of nanocarriers can be programmed to spontaneously assemble nucleic acid shells that simultaneously carry imaging labels, targeting ligands, and therapeutic payloads 105-107. The core advantage of this method is that functional complexity is encoded at the sequence level rather than added additionally afterward.

Cascade hybridization and super-sandwich strategies translate this logic into practice. Precisely engineered, programmable DNA building blocks autonomously assemble into dense polymeric shells on nanoparticle surfaces, with targeting aptamers and drug-loading domains pre-specified by sequence design. The AS1411-modified system goes a step further: ATP levels within tumor cells trigger structural reconfiguration and drug release, thereby making the nanoarchitecture itself act as a molecular sensor 34, 108. This is qualitatively different from conventional drug carriers, in which stimulus responsiveness requires separate engineering. As shown by Wang *et al.* 28, metal-ion coordination achieves comparable integration via a single-step process, with the coordination shell simultaneously loading chemotherapeutic cargo and resisting biothiol-mediated ligand displacement and nuclease digestion, as shown in Figure 3D.

Self-assembled SNAs show their clearest advantage in functional autonomy during assembly, not only after completion. As shown by Ding *et al.* 109, when siRNA is embedded as a precisely defined structural crosslinker within polymer-brush networks, the gene-silencing payload is protected by the assembly process itself, without the need for transfection reagents. Zhang *et al.* 110 developed a quantum dot-based system assembled via cascade primer exchange at room temperature. Further, they advanced it by achieving attomolar sensitivity to the lncRNA MALAT1 at single-cell resolution.

The Au-S bond remains a de facto weak link. Thiol exchange reactions and nuclease activity in physiological environments can disrupt its structural stability. A hierarchical strategy of metal-ion-triggered coordination followed by hybridization is adopted to directly address this issue, which is also the most reliable short-term approach currently available and can yield SNAs with genuine clinical biostability.

#### 2.2.3. Liposomes

As a well-established drug delivery vehicle, liposomes exhibit good biocompatibility and are capable of encapsulating hydrophilic substances 111-113. The composition of the lipid bilayer influences several key functional parameters, including DNA loading capacity, cellular uptake efficiency, serum stability, and lymph node accumulation. The aqueous core provides a fixed space for the synergistic delivery of tumor antigens and immunostimulants. Systematic optimization of these two design dimensions forms the structural foundation of the precision-engineered liposomal spherical nucleic acid (L-SNA) system.

L-SNA utilizes the encapsulation capacity of the liposome core to enable the simultaneous delivery of antigens and adjuvants. Callmann *et al.* 29 established design principles for L-SNA by modifying the composition of the liposomes; these modifications directly influenced DNA loading, cellular uptake, serum stability, and lymph node accumulation. In a triple-negative breast cancer (TNBC) model, DPPC-based L-SNAs demonstrated superior stability, reduced lung metastasis, and delayed tumor growth. In a subsequent study, Callmann *et al.* 30 encapsulated TNBC lysates into liposomes and modified their surfaces with CpG-1826 to prepare lysate-loaded SNAs. These SNAs facilitated efficient co-delivery to immune cells and inhibited tumor growth in *in vivo* models. The use of oxidized lysates further enhanced dendritic cell activation, increased CD8⁺ T cell infiltration, reduced myeloid suppressor cells, induced robust antitumor immunity, prolonged survival, and conferred resistance to re-challenge.

A customized design strategy for L-SNA based on the TME offers a viable approach to precision cancer therapy. To achieve therapeutic precision for different tumor types and promote the clinical translation of liposomal SNAs, systematic screening of phospholipid combinations and the use of microenvironment-responsive elements are indispensable.

#### 2.2.4. Peptides

Peptides occupy a special position in SNA scaffold materials, and their biological functions are encoded at the sequence level, which binds structural design to the development of therapeutic procedures 114, 115. Such inherent characteristics set them apart from inorganic or synthetic polymer carriers, for which surface modification and carrier core composition are typically performed separately.

The sequence of methionine-cysteine dipeptide-based SNAs itself can carry precise therapeutic significance. As shown by Jiang *et al.* 31, in a methionine-cysteine dipeptide-based SNA, co-loading a photosensitizer and miR-122 precisely modulated methionine metabolism, thereby reshaping the tumor immune landscape by promoting T-cell infiltration and achieving over 98% growth inhibition in hepatocellular carcinoma models, as shown in Figure 3E. The scaffold used here not only serves a structural function; its amino acid composition is precisely designed to determine the biological activity of the entire system. A zwitterionic polypeptide coating with a tumor protease recognition sequence was designed by Zhang *et al.* 83. Matrix metalloproteinases can specifically cleave this sequence, enabling the formulation to switch between circulatory stability and tumor-specific activation dynamically.

The membrane-penetrating ability of peptides can also enhance delivery efficacy. Ma *et al*. 37 modified the R11 peptide on the surface of SNAs, enabling the carrier to target bladder cancer more precisely, evade lysosomes, release the SNAs, and enhance the clarity of fluorescent imaging. The chemical properties of peptide-nucleic acid conjugates directly influence their therapeutic efficacy. Skakuj *et al.* 116 found that trace-free linkers can increase T-cell proliferation rates by a factor of 8, highlighting the critical role of conjugation chemistry in vaccine development. Related studies have confirmed that peptides possess unique advantages in SNA design due to their structural diversity, biological activity, and editability.

Peptide-based SNAs can incorporate multiple independently tunable design parameters within a single biomolecular scaffold. Constructing compound libraries by systematically modifying the cell-penetrating domain, enzyme-responsive sequences, and conjugation modes is essential for translating this flexibility into clinically applicable personalized therapies.

SNA carriers achieve multifunctional integration and enhance membrane penetration through TME-responsive release, self-assembly, and co-delivery strategies. At present, there are still multi-dimensional key issues: insufficient synergistic effect of multiple stimuli, poor stability of HER2-targeted conjugates, unclear impact of skeleton modification on biodistribution, unclear association between PD-L1 co-regulation and TLR activation and immune response, and lack of coordination in multi-load release. Gold-thiol assemblies remain susceptible to competition from biological thiols, and there is currently a lack of tailored design rules and models to govern the effects of coupling parameters, thereby further restricting the rational construction of related platforms.

Looking forward, systematic screening of coupling methods and backbone modifications will clarify how structural parameters govern *in vivo* behavior. Integrated siRNA/aptamer platforms and quantitative TLR activator–response profiling will strengthen immunotherapeutic precision. Enzyme/pH dual-responsive sequential-release systems and modular peptide/enzyme-recognition libraries should be co-developed for context-adaptive delivery. Ultimately, three-dimensional quantitative models that link coupling parameters to therapeutic outcomes are essential for bridging the gap between rational design and clinical application. A comprehensive summary of sequence and structural-functional designs is presented in Table 1.

## 3. SNA-based diagnosis

Early diagnosis of tumors relies on sensitive biomarker detection; however, traditional methods have limitations in terms of sensitivity, nuclease stability, and cell permeability 117, 118. SNAs, with their high-density three-dimensional structures and exceptional cell penetration capabilities, have enabled ultra-sensitive detection at the femto- to attomolar levels, thereby seamlessly integrating *in vitro* detection with live-cell imaging techniques. This section reviews SNA-based tumor detection from two perspectives: detection platforms, including electrostatic-regulated electrochemical sensors and core-shell energy transfer-based fluorescent imaging sensors; and diagnostic biomarkers, covering ultra-sensitive detection of exosomes, miRNAs, AMACR, and telomerase via transmembrane probes and programmable signal transduction.

### 3.1. SNA-based detection platforms

#### 3.1.1. Electrochemical sensor

Electrochemical sensors can detect tumor markers and nucleic acids quickly, sensitively, and at low cost 119-123. Traditional probe architectures have many inherent limitations, which are paid for by SNA probes that rely on three physicochemical properties; however, these probes also have limitations that warrant attention.

The compact SNA assemblies enable each recognition reaction to bind a large number of electroactive substances, amplifying the redox signal to a level far beyond that achievable by linear probes. Chen *et al.* 124 employed SNA-templated silver nanoclusters that undergo direct electrochemical reduction, yielding a precision detection limit of 7.74 fg/mL for alpha-fetoprotein. In a photoelectrochemical configuration, Cu²⁺ released from SNA-templated copper nanoclusters upon acidolysis modulates photocurrent through interaction with a CdS/Ni-CAT-1 nanorod array, enabling precision carcinoembryonic antigen detection at 0.003 pg/mL 44. The DOX-modified SNA carried by the framework nucleic acid nanomachine produces a characteristic reduction peak at -0.63 V, which can be used for accurate quantitative detection of telomerase 125. Most of these systems rely on a multi-component composite structure, and the problems of their manufacturing difficulty and structural stability in physiological environments remain unresolved.

**Figure 4:**
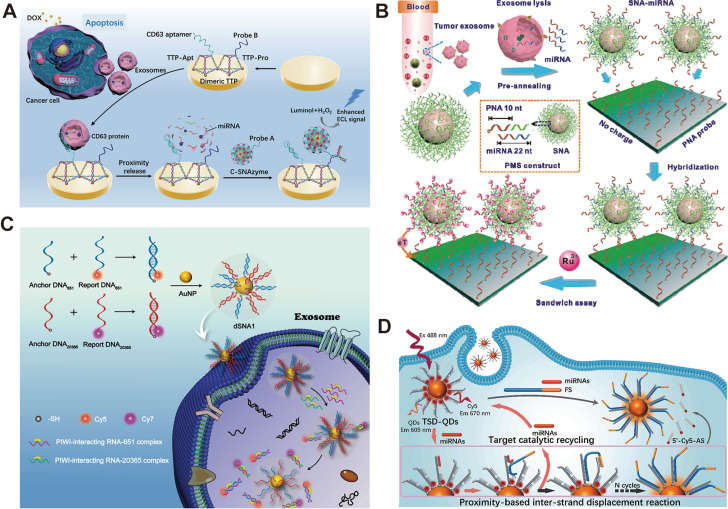
SNA-based diagnostic platform for multiplexed biomarker detection. (A) Schematic of the dimeric TTP-based ECL sensing platform for exosomal miRNA detection. Adapted with permission from 128, copyright 2023 American Chemical Society. (B) Schematic illustration of ExomiR detection using the SEEmiR sensor. Adapted with permission from 129, copyright 2019 American Chemical Society. (C) Cartoon presentation of the structure of the dual-targeted SNA probe and its mechanism of *in situ* and multiplex detection of piRNAs in exosomes. Adapted with permission from 46, copyright 2024 Elsevier B.V. (D) Schematic illustration of TSD-QDs-based amplifier for miRNA detection. Adapted with permission from 160, copyright 2022 Elsevier B.V.

The spherical geometry of SNAs prevents surface entanglement, which limits linear DNA probes, and enables sustained electron transfer at the electrode interface. Anchoring SNAs to tetrahedral DNA nanostructures confines the luminol-H₂O₂ electrochemiluminescence reaction to the immediate electrode surface, reducing diffusion losses and reaching 33 aM precision sensitivity 126. In a system designed by Miao *et al.* 127, gold nanoparticles modified with SNAs alter the probe surface density in response to target miRNAs, thereby further modulating the output signal. As illustrated in Figure 4A, Shi *et al.* 128 placed hemoglobin-modified SNAs using a double-stranded framework nucleic acid platform. This approach not only enhanced ECL efficiency but also enabled the direct detection of exosomal miR-21. Most of these geometric advantages have only been verified in strictly controlled laboratory environments, and a solution to electrode fouling in complex biological matrices has not yet been found.

SNAs carry extremely strong negative charges on their surfaces, enabling electrostatic discrimination unattainable with traditional probes. As illustrated in Figure 4B, precisely synergized neutral PNA and negatively charged SNAs: PNA minimized nonspecific electrostatic adsorption, and SNA nanoprobes maximized specific signal via electroactive tags. This precise strategy achieved a 49 aM detection limit, two orders of magnitude lower than non-SNA systems, single-base-mismatch discrimination, and clinical breast cancer diagnosis 129.

Multi-array adaptor arrays capable of simultaneously detecting proteins, miRNAs, and metabolites on a single electrode hold promise for driving further clinically valuable advancements in adaptor-based precision diagnostics. However, most current studies have only conducted proof-of-concept demonstrations for a single analyte, and clinical validation remains insufficient, which limits their current practical application.

#### 3.1.2. Fluorescent imaging sensor

Fluorescence sensing plays a key role in the detection of tumor-associated nucleic acid biomarkers 130-133. SNA-based fluorescent sensors utilize spherical nanointerfaces to enrich the analyte on the probe surface, while the core-shell structure facilitates efficient intramolecular energy transfer. These two structural features work in concert to elevate detection sensitivity to the level required for early tumor diagnosis.

The DNA strands on the surface of SNA are arranged in a highly dense and oriented manner, which can precisely regulate the distance between fluorescent molecules and gold nanocores, a feat that conventional linear probes cannot achieve at all. As demonstrated by Zhu *et al.* 45, polyA-mediated modulation of DNA loading density enables SNA-based probes to achieve a detection limit of 0.31 nM for intracellular mRNA, a 55-fold improvement over thiolated probes, with quantitative detection completed within 2 h. The same architecture confers exceptional nuclease stability, enabling reliable discrimination of differential miR-155 expression between MCF-7 and HeLa tumor cells and L02 normal cells during live-cell imaging 134. The main determinants of sensor sensitivity and diagnostic specificity are actually the interface structure, not just the chemical properties of fluorescent molecules.

The core-shell structure of SNAs can provide a spatially defined platform for resonance energy transfer. Shen *et al.* 135 developed cerasome-based SNAs embedded with dual-emission quantum dots, which are regulated by an AND-gate logic deoxyribozyme walker to precisely control the energy transfer at the core-shell interface, thereby improving imaging contrast and cancer cell recognition accuracy. At the clinical scale, SNA platforms detect miRNA at concentrations as low as 1 fM in serum, achieving 98.8% accuracy in prostate cancer miRNA profiling, demonstrating their capability for precise diagnostic applications 136. Freeze-assisted nano-scanning further eliminates the requirement for specific fluorophores or resonance energy transfer, broadening sensor design flexibility 137.

Most current SNA fluorescent sensors are designed for a single analyte type, making it difficult to capture tumor heterogeneity and establish diagnostic features based on multiple biomarkers. Future platforms integrating multi-logic-gate networks should support simultaneous detection of miRNA and mRNA to improve diagnostic accuracy and assist in the development of personalized treatment plans.

#### 3.1.3. Colorimetric sensor

In nucleic acid aptamer-based diagnostic strategies, colorimetric assays occupy a distinct position. Compared with electrochemical and fluorescence detection methods, colorimetric assays trade sensitivity for ease of use, allowing direct visual observation of results without additional instruments, making them more suitable for on-site testing and resource-scarce settings 138-140. The core question in the field at present is how much the sensitivity can be improved without losing the iconic simplicity of this method.

The color change of localized surface plasmon resonance triggered by the aggregation or dispersion of structure-specific aptamers based on gold nanoparticles is one of the most classic colorimetric detection strategies. Seo *et al.* 141 used reversibly assembled DNA nanoparticles to precisely regulate the assembly and dissociation of structure-specific aptamer probes, enabling sensitive, multi-channel detection of DNA targets without enzymatic amplification. Mollasalehi *et al.* 142 developed Au nanoprobes that target circulating miR-21 and miR-155 for the simultaneous colorimetric detection of multiple cancers. This method provides instrument-free visual readout with a detection limit below 1 ng/μL of total isolated miRNA, highlighting the potential of SNA-based assays for rapid cancer screening.

SNA immobilized on a carrier enables the G-quadruplex DNAzyme to exert colorimetric amplification via peroxidase-like activity. Shahsavar *et al.* 143 developed a G-quadruplex DNAzyme platform for the detection of miR-155. Target hybridization disrupted the DNAzyme conformation, reducing color intensity and yielding a linear response from 1 to 100 nM and a detection limit of 0.7 nM in human serum. Cheng *et al.* 144 constructed an asymmetric split DNAzyme dual-mode biosensor for detecting surface proteins on breast cancer exosomes, demonstrating the practicality of SNA-catalyzed colorimetric detection. Sai *et al.* 145 combined urease-catalyzed amplification with silver nanoparticles on an SNA scaffold to achieve ultra-sensitive nucleic acid detection, significantly expanding the dynamic range of traditional colorimetric methods and improving detection sensitivity.

Portable SNA-based detection methods are key to clinical translation. Liu *et al.* 146 integrated protease-responsive transcription with SNA polymerization into a smartphone system, enabling the detection of MMP-2 at concentrations as low as 3.3 pM. Immobilizing SNA on a glass fiber membrane allows for sensitive detection of clinical samples, thereby demonstrating the feasibility of SNA-based point-of-care testing.

A key challenge for the future is to scale this method up to multiplex lateral flow or paper-based detection systems, enabling the simultaneous detection of ctDNA, miRNAs, and protein biomarkers in non-invasive samples. Although significant technical hurdles remain to achieve this goal, its clinical value cannot be overlooked.

### 3.2. Diagnostic biomarkers

#### 3.2.1. Exosomes

Exosomes carry two distinct types of molecular markers: membrane-anchored surface proteins and nucleic acids encapsulated within the vesicles. Both of these markers present unique spatial challenges during detection. The dense nucleic acid shell of the SNA complex, combined with its multivalent recognition properties, enables it to bind to both markers simultaneously without disrupting the membrane structure. This characteristic distinguishes it from traditional detection probes 147-151.

Exosomal membrane proteins can serve as natural anchor points and reaction platforms for SNA, thereby facilitating accurate detection. Wang *et al.* 152 utilized SNA to form a poly-T template via TdT-mediated *in situ* extension, thereby triggering a cascade of amplification catalyzed by exonuclease III. This method achieved a detection limit of 44 particles/μL and was capable of distinguishing colorectal cancer patients from healthy individuals. Li *et al.* 153 designed a dual-SNA synergistic recognition system targeting CD63 and nucleolin. *In situ* TdT extension and hybridization-induced gold nanoparticle aggregation enabled a visual detection limit of 45 particles/μL. Li *et al.* 154 utilized exosomal vesicles as a physical bridge between the solid substrate and SNA. This membrane served as a spatial scaffold, triggering a hybridization cascade and generating DNAzyme, with a detection limit of 50 particles/μL.

Encapsulated nucleic acid biomarkers can provide direct molecular-level information for tumor diagnosis. As illustrated in Figure 4C, Wu *et al.* 46 utilized optimized, high-density, DNA-modified dual-targeted SNA nanoprobes to achieve *in situ* multi-target detection of piRNAs in exosomes, thereby enabling the differentiation of samples from breast cancer patients without disrupting the membrane structure. Xu *et al.* 155 anchored SNAs to the exosomal membrane via two aptamers, where recognition induced gear movement and nicking-enzyme cleavage to release fluorescent signals, achieving a 20 particles/μL detection limit for glioma-derived exosomes.

Most current platforms can only perform detection independently and cannot integrate sample pretreatment and on-site signal reading, which limits their practical application value. Combining magnetic separation technology with visually identifiable colorimetric results enables these detection systems to perform real-time screening without specialized instruments, making them particularly suitable for point-of-care scenarios where the clinical value of early cancer detection is highest.

#### 3.2.2. miRNA

There are three core challenges in miRNA detection commonly encountered in tumor diagnosis, namely extremely low abundance, rapid degradation by RNases, and highly similar sequences within miRNA families 156-159. SNA nanostructures can address these three types of problems simultaneously, enabling accurate quantification of tumor miRNAs in scenarios where traditional probes cannot, and thus have become the foundation of precise diagnoses.

Ultrashort miRNAs challenge conventional detection. The three-dimensional high-density SNA probe array enables precise recognition of these sequences. Yang *et al.* 47 developed an erythrocyte membrane-based SNA interface. It achieved a 33.9 aM detection limit for miR-141 and enabled precise single-base discrimination among members of the miR-200 family. As illustrated in Figure 4D, Zhao *et al.* 160 designed a self-propelled quantum dot SNA. A specially tailored fuel strand precisely optimizes hybridization kinetics for short miRNAs, reaching a 5.8 fM detection limit. Wang *et al.* 161 used spatial matching to guide a non-enzymatic DNA circuit that exploits the short-chain characteristic of miRNA for accurate targeting. SNA geometry itself is a tunable parameter for diagnostic precision, enabling engineering of precise SNA diagnostic platforms with optimized performance.

Cellular RNases degrade miRNAs, so probes must enable rapid capture and protection with precise release for stable intracellular monitoring. Wu *et al.* 162 precisely shielded miRNA–probe complexes from nuclease degradation using a “Trojan horse” SNA encapsulation in amphoteric carriers, enabling high-fidelity cytoplasmic miRNA imaging. Duan *et al.* 163 designed a DNA-nucleated sea urchin-like SNA that precisely accelerates binding kinetics to capture miRNAs before degradation. Jiao *et al.* 164 optimized polyA length for spatial configuration, achieving faster and more precise miRNA capture with segmented polyA SNAs to suppress oncogenic miRNAs in live cells.

Exosomal membranes in plasma block the use of conventional probes for miRNA detection. Zhai *et al.* 165 used SNAs to precisely penetrate exosomes without disrupting them, enabling accurate detection of miR-1246 from 40 μL of plasma with 100% sensitivity and 92.9% specificity for breast cancer. Xu *et al.* 166 designed a polyA-mediated fluorescent SNA whose adenine–gold affinity precisely targets miRNAs in serum and urine, achieving 104.0–113.3% recovery. A three-channel chip precisely detected miR-21, miR-141, and miR-375 for clinical profiling.

Systematic evaluations of heterogeneous clinical samples remain relatively rare, and limitations in signal pathways can also affect the ability to perform simultaneous multi-index detection. To apply such precise diagnostic capabilities to routine clinical screening and tumor subtype classification, the combination of microfluidic structures and tumor-specific miRNA libraries is essential.

#### 3.2.3. AMACR

AMACR is a protein rather than a nucleic acid; therefore, it cannot serve as a direct input signal for the CRISPR-Cas nucleic acid detection system 167. This biochemical incompatibility poses a fundamental obstacle in the diagnostic process. Precise SNA analysis technology bridges this gap by acting as a programmable signal converter, translating AMACR recognition of proteins into a nucleic acid output signal compatible with downstream amplification cascades.

This conversion principle has been applied to various SNA designs to enable SNA-based precision diagnostics. Ling *et al.* 168 constructed Y-shaped DNA nanostructures immobilized on the surface of magnetic nanoparticles, thereby achieving AMACR detection based on electrochemiluminescence with a detection limit of 1.25 ng/mL. Li *et al.* 169 developed a space-restricted co-assembly system of Y-shaped probes and hairpin probes, which increased the catalytic rate of DNAzyme by 1.6-fold; by combining this with an AuAg nanocluster-MOF-5 platform, the detection limit was extended to 1.0 × 10⁻⁴ μg/mL. Li *et al.* 170 developed an ice-cream probe-based SNA architecture that combines rolling circle amplification with CRISPR/Cas12a activation, enabling ultra-sensitive quantitative detection of low-abundance AMACR.

These platforms collectively establish SNA as an effective scaffold for AMACR-targeted biosensing. However, a key limitation is that all current designs remain confined to single-biomarker systems. Clinically, the diagnosis of prostate cancer relies on a combination of biomarkers, rather than on AMACR alone. Functionalizing spatially separated sites on the SNA surface with multiple adaptors is a reasonable approach. However, such architectures present significant engineering challenges. Interference between adaptors and the reliable simultaneous quantification of multiple targets remain pressing issues that must be systematically addressed before clinical translation can be achieved.

#### 3.2.4. Telomerase

Telomerase is selectively activated in tumor cells but remains silenced in normal tissues 171, 172. This restricted activity directly leads to uncontrolled proliferation in cancer, making it a reliable molecular phenotypic marker. Due to its programmable nucleic acid structure and inherent ability to penetrate cells, the SNA platform, which leverages these properties, is capable of precisely targeting telomerase activity within complex biological environments.

This system is based on SNA and a specific technique that converts telomerase activity into a diagnostic signal that can be quantified using various detection methods. Liu *et al.* 173 combined SNA with molecular beacons to achieve tumor recognition based on telomerase activity. This method can be applied to liquid-phase detection, single-cell analysis, and in* vivo* imaging, while also accommodating the heterogeneous nature of tumors. Shen *et al.* 125 developed an electrochemical platform that integrates framework nucleic acid nanomachines with DOX-loaded SNA tags. Through primer-extension-triggered autonomous operations and chain-displacement assembly, the platform achieved sensitive detection of HeLa cells within a concentration range of 10 to 1.0 × 10⁴ cells/mL, with a limit of detection of 2 cells/mL, thereby enabling quantitative analysis in the presence of normal cells.

Most existing platforms still rely on single-marker detection, which limits diagnostic resolution in heterogeneous tumors. The combination of magnetic separation technology, microfluidic chips, and spatially controllable polyA-SNA configurations holds promise for the simultaneous quantitative detection of multiple markers, thereby advancing the development of tumor-specific molecular fingerprint databases and facilitating precise classification and personalized treatment.

SNA features a compact three-dimensional structure, high cellular uptake efficiency, and resistance to nuclease degradation, enabling the detection of tumor markers, including exosomes, miRNAs, AMACR, and telomerase, with sensitivity ranging from attomolar to femtomolar levels. Electrochemical sensing achieves this sensitivity through electrostatic regulation, while fluorescence-based systems rely on energy transfer control, thereby bridging the gap between *in vitro* detection and live-cell imaging. Currently, commonly used detection methods typically require magnetic separation steps and standardization procedures, and are limited to single-marker analysis, which restricts their application value in resolving tumor heterogeneity. Future research in this area could advance in three directions: combining miniaturized magnetic separation with microfluidics and colorimetric methods to enable real-time, device-free detection; programming poly-A SNAs using multi-logic gates to enable multiplexed quantification of biomarkers and molecular fingerprinting; and integrating SNA-based telomerase detection with drug delivery to develop image-guided therapeutic-diagnostic systems, thereby transitioning from single-modal diagnostics to intelligent multidimensional analysis and integrated diagnosis-treatment.

## 4. SNA-based therapeutics

SNAs, featuring unique three-dimensional structures, excellent biocompatibility, and customizable surfaces, serve as key nanoplatforms for precise cancer diagnosis and therapy. When functional nucleic acids are combined with multimodal strategies, such as nanostructures, they can achieve precise tumor targeting, enhance cellular uptake efficiency, and synergistically regulate immunity and gene expression 174, 175. This section focuses on drug delivery, vaccine development platforms, and multi-system tumor therapy.

### 4.1. Drug delivery of SNAs

Traditional drug delivery systems suffer from low drug loading capacity, poor stability, and insufficient cellular uptake 176-180. SNA-based delivery platforms have evolved from single-chemotherapeutic-drug carriers to co-delivery systems with immune adjuvants, as well as intelligent platforms that can respond to the TME and release nucleic acids and metal ions in a targeted manner for precise tumor therapy 72, 181-184.

Functional modification of nucleic acid shells can address the problems of poor solubility, non-specific biodistribution, and low cellular uptake of small-molecule chemotherapeutic drugs. The colloidal stability of DOX-loaded polymeric SNAs (DOX-pSNA) is improved, and its cytotoxicity in SKOV3 ovarian cancer cells is also enhanced 185. After conjugating hydrophobic paclitaxel to the thiophosphorylated oligonucleotide backbone, the drug-loading efficiency reaches 53%, while the aptamer-targeting ability is retained and drug resistance is reversed 186. An amphiphilic DNA-paclitaxel conjugate that exploits dual gene-regulatory and hydrophobic carrier functions achieved cellular entry rates 100-fold faster than free DNA via disulfide-mediated intracellular release 187. For platinum-based agents, a Pt(IV) prodrug conjugated to a MUC-1 aptamer-containing diblock DNA strand achieved 39.6% drug loading with selective tumor targeting and reduced systemic toxicity 188. Gene-chemo synergy has been achieved by co-loading a ribozyme and DOX into a DNA core-shell SNA (ApRz CS/DOX), in which TME-overexpressed miR-21 triggers ribozyme release to cleave *PLK1* mRNA 189.

Co-delivery systems must overcome pharmacokinetic disparities to achieve spatiotemporally synchronized release. Core-shell SNAs incorporating dual adjuvants (CpG ODN and monophosphoryl lipid A) with DOX achieved MMP-9-responsive release, wherein synchronized adjuvant activation enhanced immunogenic cell death and enabled synergistic chemo-immunotherapy with reduced off-target toxicity 190. SNA-like nanoparticles conjugated to paclitaxel and fluorouracil-integrated antisense oligodeoxynucleotides with fluorescent di-thioimide achieved reversal of resistance through P-glycoprotein downregulation and cooperative dual-drug release 191.

Targeted delivery and *in situ* silencing of oncogenic nucleic acids within the TME represent the frontier of SNA-based precision therapy. Gold nanoparticle-core SNAs bearing antisense shells simultaneously captured oncogenic miR-21 and miR-155, triggered DOX release, and induced aggregation-mediated photothermal effects; the resulting increase in size prolonged tumor retention, enabling synergistic gene-chemo-photothermal therapy 192. The SNA copper-ion delivery platform with a catalase core maintains the enzymatic conversion of H₂O₂ to O₂ via long-chain DNA, thereby alleviating tumor hypoxia and sensitizing cells to cuproptosis. Multivalent CpG sequences simultaneously activate TLR9-mediated immunogenic cell death and, combined with PD-L1 blockade, induce persistent immune memory 193.

SNA-based drug delivery achieves precise tumor therapy by integrating nucleic acid shell functionalization, self-assembly, and TME-responsive release to overcome poor solubility, limited loading capacity, and drug resistance. Future designs incorporating multi-target aptamers for "recognition-capture-activation" cascade mechanisms should address tumor heterogeneity and advance the precision co-delivery of chemotherapeutics, immune adjuvants, and metal ions.

### 4.2. SNA-based vaccine platform

Vaccines have opened new avenues for cancer treatment 42, 194-198. SNA-based vaccines precisely modulate adaptive immunity by co-delivering antigens and adjuvants within a spatially defined architecture, thereby generating durable, antigen-specific immune responses.

SNA vaccines induce durable immune memory in tumor therapy. In an HPV-associated TC-1 model, Wang *et al.* 199 achieved complete tumor elimination in 30% of immunized mice and protective immunity against rechallenge. Hwang *et al.* 200 developed an SNA vaccine with a CpG adjuvant and an HPV E7 peptide. In humanized mice, it enhanced IFN-γ by more than 200-fold, doubled the number of memory CD8^+^ T cells, nearly doubled killing efficiency against HPV+ UM-SCC-104, and increased dendritic cell activation by 50%.

Another clinical benefit of SNA vaccines is their synergy with immune checkpoint inhibitors. Teplensky *et al.* 43 reported that a dual-antigen SNA enhanced antigen-specific T-cell activation and proliferation compared to a single-antigen version. In tumor models, dual-antigen SNAs with specific antigen arrangement improved antitumor responses. When combined with anti-PD-1 therapy, they suppressed tumor growth and increased the number of memory T cells. To optimize these outcomes, Yamankurt *et al.* 201 developed a high-throughput screening and machine-learning approach. They synthesized approximately 1,000 candidate SNAs in 384-well plates and rapidly assessed their immunostimulatory potency, reducing the number of required tests by an order of magnitude.

SNA vaccines precisely induce durable immune memory, enhance T-cell function, and synergize with immune checkpoint inhibitors through optimized antigen-adjuvant co-delivery and spatial arrangement. The combination of multi-omics data and machine learning is expected to enable predictive modeling of immune response characteristics, help screen for personalized antigens and adjuvants, and facilitate the transition from universal vaccines to precision cancer immunotherapy.

### 4.3. SNA-based therapies for diverse tumor types

Tumor therapy confronts several challenges, including blood-brain barrier (BBB) penetration, immunosuppressive microenvironment, and multidrug resistance 202-205. Owing to their unique three-dimensional architecture and surface modifiability, SNAs enable synergistic, precise, targeted delivery, gene silencing, and immune activation, offering a novel therapeutic strategy for refractory tumors 206-211. This section reviews the application of SNAs in tumors of the central nervous, reproductive, digestive, hematological, barrier, and respiratory systems, categorized by organ system.

#### 4.3.1. Central nervous system tumors

Glioblastoma (GBM) is the most malignant brain tumor, with a median survival time of only 12 to 15 months 212-217. Such solid tumors face unique therapeutic challenges due to the presence of the BBB, which greatly limits the delivery of drugs to the lesion site. Structurally customizable SNA nanomedicines can cross the blood-brain and blood-tumor barriers without additional transfection reagents via multivalent interactions. They can integrate oncogene inhibition, regulation of the TME, and real-time therapeutic monitoring.

The BBB hinders the delivery of drugs to central nervous system tumors, which thus differ from other solid malignant tumors. Kudruk *et al.* 218 confirmed that SNAs can achieve spatiotemporally controlled delivery to the TME through multivalent interactions and immune activation, thereby precisely bypassing the BBB. For GBM with *BCL2L12* overexpression, Jensen *et al.* 78 designed gold nanoparticle-siRNA conjugates. These SNAs crossed the BBB without transfection agents, knocked down *BCL2L12* in glioma xenografts, reduced tumor burden, and prolonged survival via enhanced caspase/p53 activity. Kouri *et al.* 219, 220 targeted miR-182, which is a prognostic biomarker. miR-182-based SNAs simultaneously inhibited the expression of *BCL2L12*, *c-Met* and *HIF2A*. Intravenous administration allowed the formulation to selectively cross the BBB, diffuse into the extravascular glioma tissue, enhance the chemosensitivity of glioma stem cells, and promote their differentiation.

**Figure 5:**
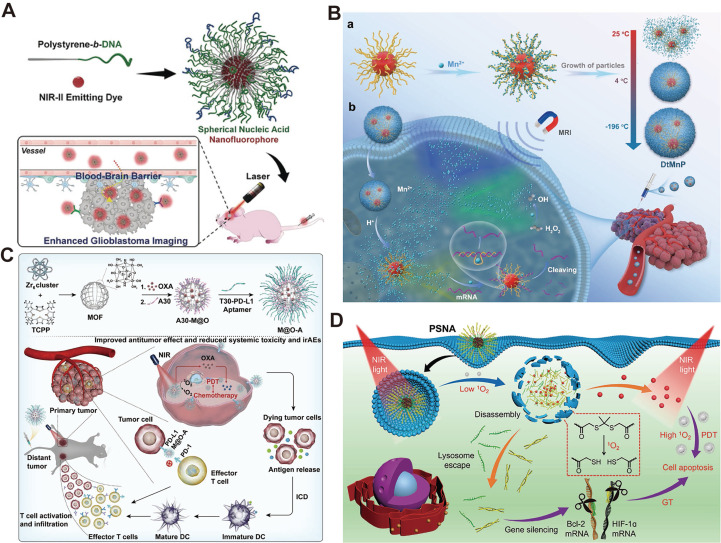
Engineered SNAs as a potent therapeutic platform for treating tumors across biological systems. (A) A DNA block copolymer-based nanofluorophore, encapsulating an NIR-II-emitting dye within a dense DNA layer, crosses the BBB via receptor-mediated transcytosis to enable non-invasive fluorescence imaging of brain tumors. Adapted with permission from 221, copyright 2020 Wiley. (B) Schematics depicting the process of synthesizing DNAzyme templated manganese nanoparticles and example diagnostic and therapeutic applications of DtMnP. Adapted with permission from 251, copyright 2025 Wiley. (C) Schematic illustration of the design and synthesis of M@O-A NPs to enable concurrent administration of photodynamic therapy, controlled release chemotherapy, and primed cancer immunotherapy with enhanced safety. Adapted with permission from 261, copyright 2022 Wiley. (D) Schematic representation of the use of PSNA to deliver siRNA, pASO, and PS for combination cancer therapy. Adapted with permission from 276, copyright 2021 American Chemical Society.

The ability of SNAs to cross the BBB can also be used for precise neuroimaging and for tracking therapeutic effects. As shown in Figure 5A, DNA block copolymer SNA delivered an NIR-II-emitting dye to GBM sites with 3.8-fold enhanced tumor localization relative to conventional agents, highlighting the superior targeting accuracy of precision SNA 221. A single intravenous injection of *MGMT*-targeted siRNA-SNAs resulted in sustained knockdown of *MGMT* in tumors. This was quantitatively validated using a complementary bimodal bioluminescence and near-infrared fluorescence imaging system, and this treatment also significantly enhanced the efficacy of temozolomide 222.

GBM chemoresistance and recurrence are driven by glioma stem cells and *MGMT*. Rouge *et al.* 223 integrated ribozymes into SNAs to cleave full-length *MGMT* mRNA. This achieved protein knockdown and sensitized GBM cells to apoptosis without the use of transfection agents. Glioma stem cells self-renew via Hedgehog signaling. Melamed *et al.* 224 designed PEI-SNAs targeting *Gli1*. Silencing this oncogene reduced *Gli1* expression by 30%, impaired self-renewal, doubled temozolomide sensitivity, and reduced metabolic activity by 60%. Given the overexpression of miR-21 in GBM, Zhu et al. 225 developed a responsive photodynamic therapy platform. The upconversion luminescence of lanthanide nanoparticles is regulated by a lock-unlock-promote strategy that inhibits reactive oxygen species generation in normal brain tissue while increasing it at tumor sites.

SNA nanomedicines can serve as carriers that cross the BBB, integrating gene therapy, imaging, and regulation of the TME to enable precise treatment of GBM. The multimodal system combining SNA-mediated gene therapy and laser interstitial thermal therapy can utilize the transient opening of the BBB during thermal therapy to synergize thermal ablation with SNA-activated antitumor immunity, converting local treatment into a systemic antitumor response.

#### 4.3.2. Reproductive system tumors

Reproductive system tumors present distinct yet interconnected challenges, including underutilized immune targets, receptor loss, drug resistance, and an immunosuppressive TME 226-233. Structurally tunable SNAs can address these issues and enable the development of precision treatment regimens tailored to the molecular characteristics of different subtypes.

SNA-based immunotherapies for prostate cancer have focused on two main strategies: antigen-adjuvant co-presentation and receptor-targeted delivery 234-237. Using PSA antigen presentation, Qin *et al.* 238 constructed immunostimulatory SNAs that simultaneously display CpG adjuvants and tumor peptides, significantly enhancing co-delivery to dendritic cells and cross-sensitization of CD8⁺ T cells. Teplensky *et al.* 239 integrated peptides derived from PSMA and TARP, which induced strong cytotoxic T-cell responses, cleared PSMA-expressing tumor cells in humanized mouse models, and directly demonstrated the regulatory role of antigen-specific structural modifications on immunogenicity. The biological characteristics of the tumor itself can also provide conditions for targeted delivery. PC-3 cells stably secrete exosomes; based on this, Alhasan *et al*. 240 encapsulated ASO nanostructures within exosomes. Leveraging the natural exosome transport pathway, this delivery method achieved an anti-miR-21 efficiency nearly 3,000 times higher than that of free ASO nanostructures. Tähtinen *et al.* 241 designed a SNA that matches the glycan pattern on the surface of prostate cancer cells and conjugated it with AON-ARV7, thereby reducing the effective uptake concentration from 120 nM to 10 nM via glycan receptor-mediated endocytosis. In summary, these studies elucidate how to systematically utilize prostate cancer-specific molecular markers to guide precise SNA-mediated interventions.

Because breast cancer exhibits molecular heterogeneity, SNA strategies must be tailored to specific subtypes 242-246. In TNBC, where receptor absence limits targetable pathways and immunosuppressive macrophage infiltration dominates the TME, ultrasound-responsive SNAs delivering *c-Myc* siRNA and PD-L1 pASO achieve simultaneous oncogene suppression, macrophage repolarization, and immune checkpoint blockade 247; systematic optimization of PEG modification demonstrates that surface-only PEGylation balances prolonged circulation with efficient cellular internalization in syngeneic models 248. Ni *et al.* 249 addressed this by using a DNA- poly(lactic acid) nanoplatform that employs *in situ* rolling-circle transcription to co-deliver DOX and an *MDR1*-targeting shRNA, thereby silencing *MDR1* and restoring drug sensitivity in resistant cells. Li *et al.* 250 exploited aberrant survivin mRNA expression in MCF-7 cells to trigger SNA-mediated delivery of miR-34a, thereby significantly reducing cell viability and inducing apoptosis. As shown in Figure 5B, Xie *et al.* 251 functionalized gold nanoparticles with DNAzymes to template pH-responsive manganese carbonate nanoparticles, enabling MRI-guided *Egr-1* silencing with 60% mRNA downregulation and Mn²⁺-catalyzed Fenton-driven apoptosis reaching 45% in MCF-7 xenografts.

Current technological advances have yet to change the status quo, in which most platforms focus on a single biomarker or molecular subtype, with inherent limitations for addressing tumor heterogeneity and the complex TME. Future research and development should prioritize modular, editable aptamer architectures that can simultaneously target multiple tumor-specific antigens and synchronously regulate related pathways, such as immune checkpoint blockade, oncogene inhibition, and TME remodeling, to fully exploit the translational value of aptamers in the treatment of reproductive system cancers.

#### 4.3.3. Digestive system tumors

Given the complex biological characteristics of digestive system tumors 252-255, SNA-based therapeutic strategies are developed based on tumor-specific molecular expression profiles to achieve precise and efficient gene immunotherapy 256. Taking colorectal cancer and hepatocellular carcinoma as examples, this section explains how the molecular characteristics of gastrointestinal tumors guide the design of SNA treatment regimens.

Colon cancer is characterized by high SR-A expression and a high incidence of distant metastasis, and it can serve as a therapeutic target based on SNA 257-259. SR-A overexpression enables specific cellular uptake of nucleic acid-modified nanoparticles; charge-reversible, coordination-crosslinked SNAs exploit DNA–SR-A interactions to co-deliver ASOs, siRNAs, and ferroptosis inducers, demonstrating strong antitumor efficacy with low systemic toxicity in CT26 models as shown by Wang *et al.* 260. Immune checkpoint blockade has been integrated through PD-L1 aptamer functionalization. As illustrated in Figure 5C, metal–organic framework nanoparticles functionalized with PD-L1 aptamers and coated with an oxaliplatin-core SNA shell synergistically integrated photodynamic, chemotherapeutic, and immunotherapeutic effects upon light activation, effectively suppressing both primary tumors and distant metastases without significant immune toxicity 261. Co-functionalization of SR-A-specific ligands with PD-L1 aptamers within a unified SNA shell represents a promising approach to enhance selective recognition of cancer cells while simultaneously addressing immune evasion and metastatic dissemination.

The liver-specific miRNA landscape of hepatocellular carcinoma offers inherent advantages for precision gene therapy 262-264, and SNA platforms that use biodegradable polycarbonate materials and an optimized cyclic topology have demonstrated particular efficacy by enabling precise co-delivery of chemotherapeutics and functional miRNAs to reverse the immunosuppressive TME 265-268. Jiang *et al.* 269 developed biodegradable polycarbonate-based SNAs co-delivering DOX and miR-122 to induce immunogenic cell death and enhance innate immunity, achieving 98.1% tumor growth inhibition *in vivo*, whereas cyclic brush-like SNA architectures exploit ultra-small size and enhanced structural stability to enable deep tumor penetration and promote neutrophil polarization, achieving 87.4% tumor suppression in comparable models as shown by Liu *et al.* 270. Key influencing factors for the therapeutic efficacy of hepatocellular carcinoma include structural tunability, encompassing material degradability and topological structure design, as indicated by existing research. Future therapeutic platforms can combine responsive materials and adaptive delivery technologies to achieve precise immune activation in different TMEs.

Despite advances in related technologies, numerous unresolved issues remain regarding the application of SNA in digestive system tumors. For example, it is difficult to adapt to the molecular differences between primary and metastatic lesions; the stability in the gastrointestinal environment remains unclear; relevant studies on gastric and pancreatic cancer are insufficient; and the mechanism of interaction with the tumor stroma has not been clearly elucidated. SNA carriers developed for the gastrointestinal axis, combined with pH- and enzyme-sensitive shells to achieve targeted drug release, and paired with microbiota-activated prodrug strategies, can better conform to the pathophysiological characteristics of digestive system tumors, providing a feasible approach for optimizing drug delivery systems.

#### 4.3.4. Hematological tumors

A typical challenge in hematological malignancies is the systemic spread of malignant cells throughout the blood and bone marrow, which not only makes complete tumor eradication difficult but also carries an extremely high risk of recurrence 271-274. SNAs, with their long circulation and lymphocyte affinity, can offer unique therapeutic advantages in such diseases, enabling sustained delivery of drugs to malignant cells in circulation, including those in lymphoma and chronic lymphocytic leukemia.

Three main therapeutic strategies have been explored so far: TLR-mediated immunomodulation, anchored chemical optimization, and combined photodynamic and gene therapy. Radovic-Moreno *et al.* 66 developed immunostimulatory SNAs that engage endosomal TLR3, TLR7/8, and TLR9 in lymphocytes, demonstrating up to 80-fold enhanced efficacy, 700-fold higher antibody titers, and 400-fold amplified cellular responses relative to free oligonucleotides, with marked survival benefits in lymphoma-bearing models. Replacing the cholesterol anchor with a dodecane (C_12_) moiety improved circulation stability. It yielded a 4-fold increase in immunostimulatory capacity, with complete resistance to tumor rechallenge observed in 50% of initial survivors, as shown by Dittmar *et al.* 275. As illustrated in Figure 5D, photostable SNAs have also been engineered to enable light-controlled silencing of the anti-apoptotic protein Bcl-2 and hypoxia-inducible factor-1α in circulating lymphoma cells, integrating gene therapy with photodynamic treatment to suppress tumor growth 276.

Within the framework of monotherapy, an integrated system that combines near-infrared-triggered, Bcl-2-targeted gene silencing with TLR-mediated immune activation will be the key direction for subsequent research and development. Such systems can achieve spatiotemporally controllable tumor suppression while activating endogenous antitumor immune responses.

#### 4.3.5. Barrier system tumors

The TME of skin cancer exhibits significant immunosuppression and complex escape mechanisms, which promote distant metastasis and limit the efficacy of immunotherapy 277-279. SNAs have been engineered to integrate antigen design with immune checkpoint blockade, leveraging localized delivery to activate both intratumoral and systemic immunity.

Spatial antigen structure is a key factor influencing immune outcomes. Teplensky *et al.* 43 showed that encapsulating helper T cell-targeted antigens inside while conjugating cytotoxic T cell-targeted antigens outside can increase the activation level of antigen-specific T cells by 30%, double their proliferation capacity, and delay tumor growth in lymphoma models; after combined treatment with anti-PD-1, the tumor suppression effect in melanoma models was enhanced, and the level of circulating memory T cells was also increased. TLR9 agonism via cavrotolimod, a 20–30 nm SNA formulated for intratumoral injection, achieved 68% inhibition as monotherapy in Merkel cell carcinoma, while co-administration with anti-PD-1 yielded 33–42% tumor suppression in squamous cell carcinoma models with favorable TME immune cell remodeling and tolerability as shown by Mix *et al.* 280.

SNA-mediated spatial antigen arrangement and innate immune activation, when combined with checkpoint blockade, can convert immunologically suppressed barrier tumors into therapeutically responsive ones. Optimizing antigen configuration and dosing regimens to maximize memory T-cell induction is a key strategy for achieving persistent systemic immune surveillance, early clearance of micrometastases, and improved long-term survival.

#### 4.3.6. Respiratory system tumors

Lung cancer initiation and progression are closely associated with the aberrant expression of tumor-promoting molecules and metastasis-related non-coding RNAs 281, 282. SNA-based therapies have been explored through two mechanistic strategies: nuclear-targeted lncRNA knockdown to suppress metastasis and siRNA-mediated gene silencing to induce cell-cycle arrest and apoptosis.

Nuclear-retained *MALAT1* is a key driver of lung adenocarcinoma metastasis. Sprangers *et al.* 60 developed a liposomal SNA platform that delivers ASOs targeting *MALAT1*, achieving dose-dependent lncRNA knockdown and concomitant upregulation of tumor-suppressor mRNAs. At the cytoplasmic level, a lipid-modified DNA vector (u4t) self-assembles into micellar SNA aggregates, with siRNA targeting the UBA6-specific E2 conjugation enzyme, that penetrate cell membranes without transfection reagents, suppressing tumor proliferation, migration, and invasion through G1-phase arrest and apoptosis *in vitro* and *in vivo*, as shown by Kim *et al.* 283.

SNAs capable of combining oncogenic transcripts from both the nucleus and the cytoplasm can inhibit multiple aspects of lung cancer progression. Future platforms should integrate TME-responsive release, nucleic acid-drug co-delivery, and image-guided monitoring capabilities. By optimizing lipid composition and surface modifications to achieve targeted accumulation in the lungs, these platforms can facilitate clinical translation.

SNAs, with their three-dimensional topological structures and high programmability, enable precise cancer therapy by facilitating stimulus-responsive drug delivery, synchronized immunotherapy-chemotherapy drug release, and sustained vaccine-mediated immunity through antigen-adjuvant synergistic delivery. Organ-specific designs can overcome the various biological barriers present in different tumor systems. Current limitations include a focus on single targets, which is insufficient to address tumor heterogeneity. Future modular, multifunctional SNA platforms should integrate multi-target, synergistic strategies, transitioning from “intelligent delivery” to “systemic regulation”, while incorporating physical modalities such as laser hyperthermia to overcome drug resistance, metastasis, and recurrence, thereby accelerating the clinical translation of precision cancer medicine. The diverse applications of SNAs across detection platforms, diagnostic biomarkers, drug delivery, vaccines, and multi-system tumor therapeutics are summarized in Table 2.

## 5. Safety evaluation

As a novel nucleic acid delivery system, SNAs demonstrate a unique and favorable safety profile across multiple levels of biological investigation 284. This chapter reviews the safety of SNA, starting with cell and animal experiments and extending to human clinical trials.

At the cellular level, finely designed SNAs exhibit almost no cytotoxicity. Ruan *et al.* 285 developed small interfering RNA delivery systems based on SNA nanostructures, including those with a DNA nanostructure core that release their cargo via Dicer-mediated cleavage, achieving effective gene silencing at the messenger RNA and protein levels while having minimal impact on cell viability. The structural precision of SNA nanostructures sets them apart from many conventional nucleic acid delivery vectors and supports their translational applications.

Through rigorous, meticulous toxicological testing, preclinical animal experiments further confirmed that SNAs offer greater safety advantages than traditional chemotherapeutic drugs. Wang *et al.* 286 constructed a bifunctional SNA nanoplatform co-loaded with siRNA and the MDM2 inhibitor NVP-CGM097, which exhibited significantly greater tolerance than melphalan in Y79 tumor-bearing mice following intravitreal injection. Kumthekar *et al.* 48 conducted systematic toxicological and toxicokinetic evaluations of NU-0129 in non-human primates and supplemented these findings with relevant research.

For therapeutic drugs based on SNA, existing human clinical trial data have clearly demonstrated a favorable safety profile, with no dose-limiting toxicities reported across studies. In a phase 0 clinical trial conducted in patients with recurrent GBM, NU-0129 was not associated with grade 4 or 5 treatment-related adverse events 48. Results from a phase 1 dose-escalation study of the TLR9 agonist cavrotolimod in healthy subjects receiving doses ranging from 5 to 18.8 μg/kg showed that all cohorts were well tolerated, with no serious adverse events reported 287. In a cohort of 58 patients with advanced skin cancer, the safety of cavrotolimod, used alone or in combination with anti-PD-1 antibodies, was manageable; a study by Milhem *et al*. 49 showed that the most common grade 3/4 adverse events were fatigue and injection-site reactions, which resolved spontaneously. These results collectively indicate that SNAs are well tolerated in clinical applications.

The findings summarized in Table 3 indicate that the favorable safety observed in these studies is associated with the pre-adjusted nanoparticle design. Core size, nucleic acid loading capacity, zeta potential, and the chemical structure of linkers/backbones simultaneously influence efficacy and tolerance. Structures with optimized surface charge and defined nucleic acid loading density can consistently achieve strong gene-regulatory effects with extremely low cytotoxicity.

Future research can focus on standardized strategies for chemical modification and structural optimization of SNAs, systematically adjusting the types and densities of nucleic acid backbone modifications further to reduce the risks of immunogenicity and long-term toxicity, and to promote the transition of such materials from the safety verification stage to reliable clinical applications.

## 6. Summary and outlook

This review summarizes the research progress on SNAs, a unique nanobiomaterial with a three-dimensional core-shell structure, in cancer therapy, covering all aspects from basic design to clinical applications. High-density radially arranged oligonucleotide shells are a typical feature of this type of structure. SNA nanostructures can efficiently enter cells without the need for transfection reagents, are not easily degraded by nucleases, and exhibit strong biological stability. These characteristics make SNAs a promising platform for addressing key shortcomings in traditional nucleic acid drug delivery.

The design strategy of SNA systems has gradually evolved from a simple gene-silencing tool to a multifunctional, integrated platform. At the sequence level, approaches such as antibody-DNA conjugation, ASOs, small interfering RNAs, and aptamers can accurately identify and regulate key tumor targets, including HER2, PD-L1, and TLR. In vector design, novel SNA structures with stimulus-responsive, self-assembling, liposomal, and polypeptide-based properties continue to emerge. In recent years, technological advances have indeed improved the controlled-release efficiency, targeting accuracy, and biocompatibility of structures such as SNA nanospheres within the complex TME.

In the field of medical applications, the scope of SNAs has expanded from their initial uses in chemotherapy drug delivery and gene silencing to cutting-edge areas such as cancer immunotherapy, vaccine development, and highly sensitive detection. In the field of drug delivery, these structures were initially capable of carrying only a single chemotherapy drug; subsequently, they were able to deliver immune adjuvants simultaneously, and today, they have evolved into smart delivery systems capable of responding to the TME. In vaccine applications, SNAs promote the formation of antitumor immune memory through spatiotemporally synchronized antigen presentation and co-stimulatory signals. When used in combination with immune checkpoint inhibitors, they can produce synergistic effects. In diagnostics, electrochemical and fluorescent sensors based on SNAs can detect key biomarkers—including exosomes, miRNAs, AMACR, and telomerase—with ultra-high sensitivity, providing new tools for early tumor screening and classification. SNAs have demonstrated encouraging therapeutic effects for various tumors affecting the central nervous, digestive, reproductive, hematological, and barrier-related systems. These agents possess enhanced cell penetration and targeting capabilities, with particularly significant advantages in crossing the BBB, reversing immunosuppression, and overcoming multidrug resistance. Preliminary preclinical and clinical studies of SNAs indicate good biosafety and tolerability, with no apparent dose-related toxicity, laying a solid foundation for clinical translation.

Although SNAs hold potential therapeutic value, the field still faces several fundamental limitations. The vast majority of relevant studies fail to systematically document key structural parameters—such as core diameter, oligonucleotide density, zeta potential, and backbone chemistry—when reporting biological experimental results, making it difficult to clearly establish a causal relationship between structure and function. Such methodological inconsistencies confine research progress to case-by-case empirical optimization rather than rational, precise design. Future research should prioritize standardized, multi-parameter characterization across different platforms under uniform experimental conditions to establish a quantitative structure-function-effect framework, thereby guiding the *de novo* design of next-generation supramolecular nanoassemblies tailored to specific therapeutic scenarios.

Despite these advances, numerous challenges remain in translating SNAs from the laboratory to widespread clinical use. The *in vivo* stability of HER2-targeted antibody-aptamer conjugates needs to be optimized; the multicellular regulatory mechanisms underlying PD-L1 targeting need to be elucidated; and a quantitative relationship between dual TLR activation and the immune response needs to be established. All of these factors indicate that drug delivery efficiency and targeting accuracy still require further improvement. Numerous practical problems must be addressed during the carrier design stage. Coordination of the release rhythm of multiple components in stimulus-responsive systems, improvement of the biological stability of gold-thiol self-assembled structures, optimization of the design specifications for liposome and polypeptide carriers, and development of quantitative models that correlate structural parameters with therapeutic effects are all key technical challenges at present.

Early-phase trials of NU-0129 and cavrotolimod have shown favorable tolerance data, yet critical manufacturing and safety hurdles remain unresolved. GMP-scale production imposes strict requirements for batch-to-batch consistency, including quantitative control of oligonucleotide surface density, endotoxin levels below 5 EU/kg/h, and a polydispersity index below 0.2. However, these standards are rarely achieved in current laboratory-scale synthesis, and relevant reports are even scarcer. Repeated injection of highly reactive nanoparticles into animals poses significant risks of immunotoxicity, including complement activation and cytokine release syndrome. Preclinical models indicate that non-degradable nanoparticle cores, such as those made of gold, can persist in the liver and spleen for months, raising concerns about chronic organ toxicity. Currently, there are no reliable long-term pharmacovigilance data available. SNAs enable precision therapy and allow for spatiotemporally controlled gene regulation targeting specific molecular targets. However, realizing this potential requires standardized characterization procedures and a comprehensive post-market regulatory framework, neither of which has been systematically established to date.

Compared to LNPs, the delivery vehicles used in approved mRNA vaccines and patisiran, SNAs exhibit greater resistance to nucleases and can be taken up by cells via scavenger receptor-mediated endocytosis, making them more suitable for local administration. However, LNPs still possess decisive advantages over SNAs in terms of liver targeting efficiency, payload capacity, and scalable production compliant with GMP standards. Compared to ASOs such as nusinersen, SNAs avoid the thiophosphate modification associated with thrombocytopenia and nephrotoxicity. However, ASOs are supported by a broader body of clinical safety data and have a simpler regulatory approval pathway. The key point is that, in the short term, SNAs are unlikely to replace established platforms in the field of systemic drug delivery. In precision medicine applications, various existing platforms typically face fundamental limitations, such as difficulty crossing the BBB, inability to modulate the local TME, and lack of the multivalent antigen presentation capabilities required for cancer vaccines, which make it difficult for them to realize true clinical value in the near term.

Future research in the field of SNA should move toward smarter, more integrated systems that place greater emphasis on individual needs. Research efforts should focus on developing intelligent platforms capable of integrating multiple targeted effects, responding to the microenvironment, and enabling combination therapy, while also featuring functions such as timed release and the integration of diagnosis and treatment. Replacing non-degradable gold cores with degradable cores based on liposomes, polymers, or proteins can reduce the risk of long-term residue and simplify the regulatory approval process. Systematic, controlled studies should be conducted to compare the differences in pharmacokinetic profiles, immunotoxicity profiles, and large-scale production potential among SNAs with cores of varying chemical compositions, thereby guiding the rational selection of platforms. By combining high-throughput screening with multi-omics data and machine learning technologies, quantitative structure-function models for SNAs can be established, thereby accelerating the development of personalized treatment regimens tailored to different patients and tumor types. Through interdisciplinary collaboration and continuous innovation, SNAs are poised to become a cornerstone technology in precision cancer medicine, facilitating a seamless transition from basic research to clinical practice.

## Figures and Tables

**Table 1:** Sequence and structural-functional architecture design of engineered SNAs.

Type	Classification	Material composition	Target	Characteristic	Application	Ref.
Sequence design	HER2	mAb recognizing the HER2, sense DNA sequence, AuNPs	HER2	Antibody-mediated targeting; Enhanced cellular uptake; Direct HER2 gene silencing	Targeted therapy	23
		β-CD, Ada, HER2 antisense nucleic acids, AS1411	HER2	Inhibition of HER2 overexpression	Targeted therapy	24
	PD-L1	PD-L1 aptamer MJ5C, HfO₂, ICG	PD-L1	High-specificity binding to PD-L1; Potentiation of radiotherapy	Targeted therapy	93
	TLR	Poly(I:C), CpG oligonucleotide, liposomes (DOPC)	TLR3, TLR9	Synergistic activation of multiple TLR pathways	Targeted therapy	40
Design of biomaterial carrier	Intelligent controlled release carrier	APE1, AP site-containing trailing DNA hairpins, DBCO-ASO, PLA-N3, DOX	survivin mRNA	Initiated by endogenous enzymes	Cancer detection	26
		Poly(deoxycytidine), HS-primer, AuNP, rG-AS1411, DOX	Nucleolin	pH-responsive system	Targeted therapy	101
		AIE photosensitizer (TBD-PEG-N₃), Bcl-2 ASO	Bcl-2 mRNA	Light-triggered release	Targeted therapy	102
	Self-assembly	Streptavidin, Biotinylated hairpin probes, QDs, dumbbell probe targeting lncRNA (MALAT1), KF Polymerase	lncRNA MALAT1	Biomolecular interaction-mediated	Cancer detection	110
	Liposomes	Liposomes (DOPC, DMPC, DPPC, DSPC), CpG 1826 (a murine TLR9 agonist), tumor cell lysates	TLR9	Modulation of membrane order by lipid tail structure	Targeted therapy	29
	Peptides	EK polypeptide, MMP-cleavable peptide linker, liposome (DOPC), ODN	MMP-9	Antifouling-and-biocompatible zwitterionic peptide	Targeted therapy	83

Abbreviations: Ada: adamantane; AIE: aggregation-induced emission; APE1: apurinic/apyrimidinic endonuclease 1; ASO: antisense oligonucleotide; AuNP: gold nanoparticle; β-CD: β-cyclodextrin; CpG: cytosine-phosphate-guanine; DBCO-ASO: dibenzocyclooctyne-modified antisense oligonucleotide; DOX: doxorubicin; E: glutamic acid; HfO₂: hafnium oxide; ICG: indocyanine green; K: lysine; KF: Klenow fragment; mAb: monoclonal antibody; MALAT1: metastasis-associated lung adenocarcinoma transcript 1; MMP-9: matrix metalloproteinase-9; ODN: oligonucleotide; PLA-N3: azide-containing aliphatic polymer poly(lactic acid); QDs: quantum dots.

**Table 2:** Different applications of SNAs.

Type	Object	Material composition	Target	Characteristic	Application	Ref.
Detection Platform	Electrochemical Sensor	DOX, AuNPs, a double helix of DNA	Telomerase	Intense electrochemical signal with powerful amplification	Cancer detection	125
	Fluorescent imaging sensor	6-carboxyfluorescein-labeled DNA, AuNPs, backbone-modified PS DNA	miR-30a	Versatile detection platform using fluorescent dyes	Cancer detection	137
	Colorimetric Sensor	AuNPs, thiol-modified DNA probe, miR-21	miR-21	Colorimetric readout based on non-crosslinking aggregation, visual detection with the naked eye	Cancer detection	142
Diagnostic biomarker	Exosomes	Apt-CD63, Apt-AS1411, multifunctional probes, AuNPs	Glioma exosomes	High specificity for glioma-derived exosomes	Cancer detection	155
	miRNA	Hairpin probes H1 and H2, UDNs	miR-21	Non-interfering biocompatibility for accurate miRNA detection	Cancer detection	163
	AMACR	AMACR aptamer, gold-magnetic nanoparticles (Au@Fe₃O₄), IC probes, DNA walker, APE1	Prostatic AMACR	High specificity and sensitivity in AMACR detection	Cancer detection	170
	Telomerase	TP-carrying strand, FL-strand, AuNPs	Human telomerase	Cross-platform telomerase activity detection in cancer cells	Cancer detection	173
Delivery	Chemotherapeutic agents	DOX, AuNPs, full anti-miR-155 sequence, half the anti-miR-21 sequence	miR-155, miR-21	Stable drug loading with minimal circulatory leakage	Drug delivery	192
	Metal ions	copper ions, CAT, poly(CpG), DBCO-labeled primers	TLR9	Robust protection against premature Cu²⁺ leakage and deactivation	Drug delivery	193
	Immune adjuvant	MPLA, CpG ODN, DOX, sMMP9	TLR9, TLR4	Co-delivery of dual adjuvants for synergistic activation	Drug delivery	190
Vaccine	Multivalent vaccine	MHC-I-restricted and MHC-II-restricted, CpG motif adjuvant DNA, liposomes (DOPC)	TLR9	Dual cytotoxic and helper T cell pathway activation via multi-antigen targeting	Immunotherapy	43
	Monovalent vaccine	Peptide antigen (E711-19), CpG 1826, liposomes (DOPC)	TLR9	Enhanced immunogenicity of mono-antigen via rational design	Immunotherapy	200
Therapeutics	Central nervous system tumors	CpG ODN, AuNPs	TLR9	High capability for BBB penetration	Targeted therapy	218
	Reproductive system tumors	Peptide antigen (PSMA711–719, TARP 29-37-9V), DOPC, CpG DNA	PSMA, TARP	Precise targeting of highly expressed prostate cancer-specific targets	Targeted therapy	239
		c-Myc siRNA, PD-L1 pASO, PpIX	PD-L1 mRNA	Combined modalities against TNBC for synergistic efficacy	Targeted therapy	247
	Digestive system tumors	siRNA, poly(lactic acid), ASO, PEG, imidazole, DBCO	Scavenger receptor-A	Precise targeting of colorectal cancer cells and tissues, tailored to pathological features	Targeted therapy	260
		miR-122, c-P(HEMA)	p-STAT3	Cyclic brush topology with high stability and efficient miR-122 delivery for HCC	Targeted therapy	270
	Hematological tumors	pASO targeting Bcl-2, HIF-1α siRNA, PPa	Bcl-2 mRNA	Adapted to the immune-sensitive hematological state with favorable biocompatibility and low immunogenicity	Targeted therapy	276
	Barrier system tumors	Four TLR9-agonizing CpG motifs, DOPC, a cholesteryl ester moiety	TLR9	Local drug delivery and lesion targeting adapted to barrier-system tumors	Targeted therapy	280
	Respiratory system tumors	ASOs, liposomes (DOPC), phosphorothioate, PO	MALAT1 lncRNA	Targeting nuclear-localized oncogenic drivers in respiratory system tumors	Targeted therapy	60

Abbreviations: ASO: antisense oligonucleotide; CAT: catalase; c-P(HEMA): cyclic poly(2-hydroxyethyl methacrylate); DBCO: dibenzocyclooctyne; DOPC: 1,2-dioleoyl-sn-glycero-3-phosphocholine; FL: fluorophore; IC probes: ice-cream probes; MPLA: monophosphoryl lipid A; pASO: peptide nucleic acid-based antisense oligonucleotide; PO: phosphodiester; PPa: pheophorbide a; PpIX: protoporphyrin IX; PSMA: prostate-specific membrane antigen; sMMP9: substrate peptide of matrix metalloproteinase-9; TARP: T-cell receptor γ alternate reading frame protein; TNBC: triple-negative breast cancer; TP: telomerase primer; UDNs: urchin-like DNA nanostructures.

**Table 3:** Design parameters of SNAs and their impact on efficacy and toxicity.

Core size	Nucleic acid density	Zeta	Linker/Backbone	Efficacy/Toxicity	Ref.
30 nm liposomal cores	On average, 70 DNA strands per particle	-1 to -23 mV	DOPC lipid monomer, DNA strands modified with a tocopherol tail	HER2 protein levels reduced by 85%, no measurable toxicity	57
15 nm gold nanoparticles	38 siRNA duplexes per particle	+27 mV	Radially oriented siRNA and poly(ethylenimine)	Enhanced gene silencing potency, decreased toxicity per PEI content	288
20.9 nm silica-encapsulated iron oxide nanoparticles	18 dsDNA strands per nanoparticle	-36.6 mV	Silica shell with DBCO/azide click chemistry	Full gene expression recovery, clinically tolerable field frequency	231
100 to 200 nm native EVs	3453 ± 206 DNA strands per vesicle	-15.2 to -21.2 mV	Cholesterol-modified oligonucleotides	10-20 times enhanced delivery; ≈17.6-fold higher uptake; excellent biocompatibility; no significant effect on cell viability	147
36.0 ± 4.3 nm PLGA nanoparticles	Surface density of 13.5 ± 0.7 pmol/cm² for NaCl SNAs	-40.5 ± 2.1 mV	Poly(lactic-co-glycolic acid) (PLGA) core with DBCO-modified siRNA duplexes	Up to a 20-fold enhancement in gene regulation activity, no apparent toxicity	289

Abbreviations: DOPC: 1,2-dioleoyl-sn-glycero-3-phosphocholine; DBCO: dibenzocyclooctyne; EVs: extracellular vesicles; HER2: human epidermal growth factor receptor 2; PEI: polyethylenimine; PLGA: poly(lactic-co-glycolic acid).

## Abbreviations

Ada: adamantane
AIE: aggregation-induced emission
AMACR: alpha-methylacyl-CoA racemase
AP: apurinic/apyrimidinic
APE1: apurinic/apyrimidinic endonuclease 1
ASO: antisense oligonucleotide
AuNP: gold nanoparticle
BBB: blood-brain barrier
CAT: catalase
CpG: cytosine-phosphate-guanine
c-P(HEMA): cyclic poly(2-hydroxyethyl methacrylate)
DBCO: dibenzocyclooctyne
DBCO-ASO: dibenzocyclooctyne-modified antisense oligonucleotide
DOPC: 1,2-dioleoyl-sn-glycero-3-phosphocholine
DOX: doxorubicin
E: glutamic acid
EC: electrochemical
ECL: electrochemiluminescence
EVs: extracellular vesicles
FL: fluorophore
Fluo: fluorescent
GBM: glioblastoma
HER2: human epidermal growth factor receptor 2
HfO₂: hafnium oxide
IC: ice-cream probes
ICG: indocyanine green
K: lysine
KF: Klenow fragment
LNP: lipid nanoparticle
L-SNA: liposomal spherical nucleic acid
mAb: monoclonal antibody
MALAT1: metastasis-associated lung adenocarcinoma transcript 1
MGMT: O^6^-methylguanine-DNA methyltransferase
MMP-9: matrix metalloproteinase-9
MPLA: monophosphoryl lipid A
ODN: oligonucleotide
pASO: peptide nucleic acid-based antisense oligonucleotide
PD-L1: programmed death-ligand 1
PEG: poly(ethylene glycol)
PEI: polyethylenimine
PLA-N3: azide-containing aliphatic polymer poly(lactic acid)
PLGA: poly(lactic-co-glycolic acid)
PO: phosphodiester
PS: photosensitizer
PPa: pheophorbide a
PpIX: protoporphyrin IX
PSMA: prostate-specific membrane antigen
QDs: quantum dots
sMMP9: substrate peptide of matrix metalloproteinase-9
SNA: spherical nucleic acid
SR-A: scavenger receptor-A
TARP: T-cell receptor γ alternate reading frame protein
TCO: trans-cyclooctene
TLR: toll-like receptor
TME: tumor microenvironment
TNBC: triple-negative breast cancer
TP: telomerase primer
UDNs: urchin-like DNA nanostructures
β-CD: β-cyclodextrin
